# Interhemispheric Cortical Inhibition Is Reduced in Young Adults With Developmental Coordination Disorder

**DOI:** 10.3389/fneur.2018.00179

**Published:** 2018-03-23

**Authors:** Jason L. He, Ian Fuelscher, Peter G. Enticott, Wei-peng Teo, Pamela Barhoun, Christian Hyde

**Affiliations:** ^1^Deakin Child Study Centre, School of Psychology, Deakin University, Geelong, VIC, Australia; ^2^School of Exercise and Nutrition Sciences, Institute for Physical Activity and Nutrition (IPAN), Deakin University, Geelong, VIC, Australia

**Keywords:** developmental coordination disorder, motor control, cortical inhibition, transcranial magnetic stimulation, movement disorders, movement, interhemispheric connectivity

## Abstract

**Introduction:**

While the etiology of developmental coordination disorder (DCD) is yet to be established, brain-behavior modeling provides a cogent argument that neuropathology may subserve the motor difficulties typical of DCD. We argue that a number of the core behavioral features of the DCD profile (such as poor surround inhibition, compromised motor inhibition, and the presence of mirror movements) are consistent with difficulties regulating inhibition within the primary motor cortex (M1). This study aimed to be the first account of the integrity of cortical inhibition in motor cortices in DCD.

**Method:**

The sample consisted of eight adults with DCD aged (18–30 years) and 10 aged matched neurotypical controls. Participants received a common battery of single and paired-pulse transcranial magnetic stimulation from which a series of neurophysiological measures classically used to measure intra- [e.g., short-interval cortical inhibition (SICI), long-interval cortical inhibition (LICI), and cortical silent period] and inter hemispheric [e.g., ipsilateral silent period (ISP)] cortical inhibition of the M1 at rest were recorded.

**Results:**

While no group differences were observed for any measure of intrahemispheric cortical inhibition, individuals with DCD demonstrated significantly reduced interhemispheric cortical inhibition relative to controls, shown by consistently lower ISP_ratios_.

**Conclusion:**

Our findings are consistent with the view that regulation of cortical inhibition of M1 activity may be atypical in individuals with DCD, indicating differential GABAergic operation. This effect, however, appears to be select to cortical inhibition. Importantly, our data support the notion that reduced interhemispheric M1 cortical inhibition may at least partly explain commonly reported difficulties with bimanual motor control in DCD. The neurochemical implications and limitations of this evidence will be discussed.

## Research Highlights

- A number of core behavioral features of DCD are consistent with difficulties regulating GABAergic activity (*viz* cortical inhibition) within the primary motor cortex (M1).
- This study used TMS to probe the integrity of cortical inhibition in the M1 in individuals with DCD.
- While intrahemispheric M1 cortical inhibition appears to be preserved in DCD, interhemispheric M1 cortical inhibition was significantly reduced relative to typically developing individuals.
- Our study is the first to provide evidence that regulation of GABAergic activity within the M1 may be atypical in DCD.

## Introduction

Developmental coordination disorder (DCD) is characterized by atypical development and performance of motor skill in the absence of any identifiable neurological or medical conditions (1). Diagnosed individuals present with motor skills substantially below that expected given their age, limiting their ability to perform daily tasks requiring coordinated movements [e.g., using utensils, playing sports, and self-care (2, 3)]. Motor difficulties symptomatic of DCD emerge in childhood, with the syndrome diagnosed early in development, typically during the primary school years (4). Initially theorized to result from a delay in brain development [e.g., minimal brain dysfunction: see (5) for a good review], the primary symptoms of DCD were expected to dissipate with age (6). It is now well understood that the motor skill difficulties seen in DCD persist into adulthood for a substantial proportion of diagnosed individuals (7–9), as do the secondary psychosocial difficulties (10, 11). These findings highlight the importance of early identification of DCD and the need to develop age-appropriate assessment and therapeutic strategies.

While the etiology of DCD remains unclear, brain-behavior modeling provides a cogent argument that cortical neuropathology may subserve atypical motor development (5, 12–14). Indeed, *in vivo* neurological measurement of individuals with DCD has increased substantially over the last decade (14). These studies have primarily focused on the pattern of cortical de/activation (*via* fMRI or EEG) when individuals with DCD perform perceptuo-motor and cognitive tasks, or the structural integrity of motor circuitry (*via* MRI). While promising, these data remain sparse and inconsistent. Accordingly, the “neural signature” of DCD is yet to be established (5), and further work is required. In this study, we investigate whether atypical regulation of the nervous system’s principle inhibitory neurotransmitter, gamma-aminobutyric acid (GABA), within the primary motor cortex (M1) may explain some of the core features of the DCD symptom profile (see below). To the best of our knowledge, no study has directly tested this important hypothesis nor, have the neurochemical properties of poor motor control in DCD been reported on. Since regulation of M1 activity has been shown to be amenable to therapeutic intervention in populations with and without motor impairment (15–18), clarifying this issue holds considerable therapeutic significance for individuals with DCD.

### Primary Motor Cortical Inhibition Is Fundamental to Motor Control

The M1 is the final relay point for outgoing motor commands before they descend the spinal cord, receiving input from other motor cortical and subcortical structures (19). The role of M1 in producing movement is well established, with excitation increasing within the contralateral M1 immediately (i.e., ≈120 ms) before activation of task-relevant peripheral muscles (19, 20). However, it is becoming increasingly clear that inhibitory mechanisms within the M1 are equally as important to producing mature movement (19–21). That is, accurate motor output from the M1 is part the product of excitatory commands supporting the movement of choice and part inhibitory commands suppressing undesired muscle activity (22).

Intracortically, inhibitory interneurons within the contralateral M1 are involved in the suppression of proximal, task-inappropriate muscles during voluntary manual movement (21, 23–26). Specifically, before (and during) unimanual movement, GABAergic activity decreases for task-relevant muscles, yet simultaneously and selectively increases for nearby task-irrelevant or synergistic abductors (27–29). This so-called “surround inhibition” is touted to be the putative neurophysiological mechanism that focuses, or “sharpens,” outgoing motor commands from the M1 (22, 30). Indeed, where surround inhibition is reduced, high precision manual performance is often compromised [e.g., focal hand dystonia and Parkinson’s disease (22, 31)]. Furthermore, just as regulation of intracortical M1 inhibition is central to the fine tuning of complex action, it is also critical in those instances where the nervous system is required to terminate either a prepared or prepotent action (as per the Stop-signal task and the Go/No-go task). Case in point, intrahemispheric cortical inhibition within the M1 non-selectively increases (i.e., ≈160 ms) before the successful, sudden cancelation of prepared or prepotent actions (25, 32, 33), a process known as motor response inhibition (34).

Finally, during the performance of common unilateral movements, the nervous system has a natural tendency toward activating homologous muscles in the ipsilateral limb (35, 36). In these instances, interhemispheric cortical inhibition is required to prevent contralateral M1 activity traversing the corpus callosum to the ipsilateral M1, and the subsequent (unwanted) replication of action, or part thereof, in the ipsilateral hemisphere, known as “mirror movements” (35, 37, 38). Indeed, reduced interhemispheric cortical inhibition has been reported in a number of patient groups where “mirror movements” are common (39–42).

In short, efficient and accurate motor control is predicated on the nervous system’s capacity to regulate M1 cortical inhibitory mechanisms. Given this, it is perhaps unsurprising that there is a growing body of evidence suggesting that the integrity of M1 cortical inhibition may predict the quality of motor performance (43). For example, stronger intrahemispheric cortical inhibition regulation in the M1 at rest predicts better motor performance in neurotypical adults (44). Conversely, reduced intrahemispheric cortical inhibition within the M1 is associated with atypical motor function in patients with neurodevelopmental disorders [e.g., ADHD (45)], neurodegenerative diseases [e.g., Parkinson’s disease (46, 47) and Huntington’s disease (48, 49)] and neurological conditions where motor control is compromised [e.g., focal hand dystonia (30, 50, 51) and Tourette’s syndrome (52)].

### Transcranial Magnetic Stimulation (TMS): Insight Into Intrahemispheric and Interhemispheric Cortical Inhibition of the PMC

Transcranial magnetic stimulation of the M1 has seen widespread use as a non-invasive means of investigating intra- and interhemispheric GABAergic activity (53, 54). A single-magnetic pulse delivered by TMS to the M1 in humans can produce activation in contralateral peripheral muscles. This muscle activation, also known as a motor-evoked potential (MEP), can be measured by electromyography (EMG) electrodes. With respect to intrahemispheric cortical inhibition, paired-pulse TMS (ppTMS) and cortical silent period (CSP) protocols are the most commonly adopted investigative methods (55, 56). ppTMS involves delivering two pulses to the same cortical point within the M1 in quick succession. The first pulse is a subthreshold “conditioning” pulse, which is then followed closely by a second suprathreshold “test” pulse. Depending on the time between the pulses, or “interstimulus interval” (ISI), a suppression of the muscle response is observed and is thought to reflect activity of GABAergic receptors within the M1 (57). Shorter ISIs (i.e., 2–5 ms) between pulses are thought to activate fast-acting GABA_A_ receptors (58), a protocol referred to as short-interval cortical inhibition (SICI). Conversely, longer ISIs (i.e., 100–150 ms), where both conditioning and test pulses are suprathreshold, are thought to activate relatively slower-acting GABA_B_ receptors (59), a protocol referred to as long-interval cortical inhibition (LICI). With respect to CSP, a single-TMS pulse is delivered to the cortical point on the M1 during voluntary muscle activation of the relevant peripheral muscle in the contralateral effector (60, 61). Generally, a period of suppressed muscle activity known as the “silent period” can be observed immediately following an initial TMS elicited MEP burst. CSPs are also thought to reflect GABA receptor activity (62); however, the nature of the receptor site is still a source of debate.

Finally, TMS procedures can also be reliably applied to index the integrity of interhemispheric cortical inhibition (63–65). While the latter can be inferred using various techniques, one of the more common is *via* the measurement of ipsilateral silent periods [ISPs (64, 66–68)]. Here, a single-TMS pulse is delivered to the M1 on the ipsilateral side of an active muscle, while the contralateral homologous muscle is at rest. When an MEP is elicited in the contralateral hand as a result of the TMS pulse, a brief “silent period” in muscle activity can be observed in the homologous ipsilateral muscle. ISP is widely accepted as one of the TMS-evoked measures that reflect the brain’s capacity to prevent transcallosal communication of motor commands (69–71), restricting neural activity to the contralateral M1 during unilateral movements and preventing unwanted mirror movements (35). Like measures of intrahemispheric cortical inhibition, interhemispheric cortical inhibition is also thought to be dependent on both GABA_A_ and GABA_B_ receptor activity (72).

### M1 Cortical Inhibition and DCD

As outlined earlier, there is compelling evidence indicating that the efficiency with which M1 cortical inhibition is regulated predicts motor ability [e.g., Ref. (44)]. Indeed, reduced cortical inhibition within the M1 predicts compromised motor function in neurodevelopmental disorders that commonly co-occur with DCD, such as ADHD (45). Despite this, no study to our knowledge has directly investigated the integrity of cortical inhibitory processes in DCD, a disorder primarily characterized by impaired motor control.

There are, however, several converging lines of evidence that support the view that cortical inhibition of the M1 may be compromised in DCD. Recent experimental work is replete with evidence that individuals with DCD present with behavioral phenotypes that are traditionally associated with reduced intra- and interhemispheric cortical inhibition. For example, a number of studies have shown that individuals with DCD demonstrate an increased incidence of the type of “mirror movements” symptomatic of poor interhemispheric inhibition (73–77). Likewise, children with DCD have consistently shown difficulties performing tasks that require the sudden cancelation of both prepared or prepotent movements (78–87). Since motor response inhibition is dependent on efficient intrahemispheric cortical inhibition within the M1 (25, 33), poor performance could feasibly occur due to poor modulation of intrahemispheric GABAergic activity. It should also be noted that individuals with atypical motor skills often show poor performance on various neuropsychological tests of inhibition (88). However, it is often difficult to discern whether such deficits are motoric or cognitive in nature given the cognitive demands of these tasks (89), the latter of which are unlikely to be rooted in the M1. Finally, while the inefficient, slow, and highly variable performance during complex manual tasks typical of individuals with DCD is unlikely to have a single-neurological correlate, it is nonetheless consistent with reduced surround inhibition. Again, surround inhibition is a process dependent on selective upregulation and deregulation of cortical inhibition within the M1 for the purpose of “fine tuning” motor commands (22). Taken together, we argue that the clinical and experimental profile of individuals with DCD is consistent with pathophysiology of GABAergic activity within the M1, which warrants investigation.

### The Present Paper

The aim of this study was to investigate the integrity of intra- and interhemispheric cortical inhibition within the M1 in a sample of young adults with DCD using TMS. Similarly to recent work, given the heterogeneity associated with samples of individuals with DCD, we opted to include young adults with DCD rather than children to control for the subgroup of children who “out-grow” the condition thereby reducing one common source of variability (90, 91). Given the exploratory nature of this study, M1 cortical inhibition was measured using a battery of single-pulse TMS and ppTMS protocols commonly adopted to investigate intra- and interhemispheric cortical inhibition. Based on evidence that cortical inhibition within the M1 predicts motor function in neurotypical and atypical populations, and evidence that individuals with DCD regularly present with behavioral markers consistent with atypical regulation of GABAergic mechanisms in the M1, it was predicted that individuals with DCD would present with reduced intra- and interhemispheric cortical inhibition relative to controls.

## Materials and Methods

### Participants

The sample comprised 8 adults with DCD (4 males and 4 females, M_age_ = 23.75, SD = 1.67, age range = 21–32) and 10 typically developing (TD) controls (6 males and 4 females, M_age_ = 26.00, SD = 4.24, age range = 21–26). All participants self-reported being right handed, gave written informed consent, and were screened to ensure they were free of TMS contraindications. No participants reported taking medication that would contraindicate TMS, and none reported experiencing any negative side-effects during or following TMS. The project received ethical clearance from Deakin University Human Research Ethics Committee.

Participants were recruited through advertisements placed on university websites at an Australian University and social media outlets (i.e., Facebook), targeting both individuals with motor difficulties and typical motor skill. All participants were screened using methods that have been successful by ours and other research groups in identifying adults with DCD (90–94). Prospective participants first completed the Adult Dyspraxia/Developmental Coordination Checklist [ADC (95)], and those who were deemed eligible then had their motor ability assessed using the Bruininks–Oseretsky Test of Motor Proficiency, Second Edition [BOT-2 (96)]. The BOT-2 is a well-validated standardized measure of motor skill, containing subtests that index each participant’s “Fine manual control,” “Manual coordination,” “Body coordination,” and “Strength and agility.” Based on each participant’s performance on each of the subtests, “Total Motor Composite” scores (M = 50, SD = 10) are then generated for each participant, providing an index of their age-normed motor ability. The BOT-2 was adopted over other standardized measures of motor ability based on recent reviews, which found the BOT-2 to be the most valid and reliable battery available for identifying motor difficulties in young Australian adults (97, 98).

All participants with DCD were selected according to DSM-5 criteria and in accordance with recent guidelines for identifying DCD in adults (99). Participants were considered to have DCD if their motor proficiency was significantly below that expected given their age (Criterion A), as indicated by BOT-2 “Total Motor Composite” scores at or below the 15th percentile (14). In total, 13 participants met this criterion. These motor difficulties must have significantly impacted their ability to undertake daily activities involving movement (Criterion B) and arose in childhood (Criterion C), as determined using the ADC. The ADC is a 40-item questionnaire designed to identify current and childhood difficulties with completing daily living tasks related to motor function. The ADC is often used to determine whether poor motor skill is associated with a reduced ability to perform tasks of everyday living (93, 100–102). While ideal for addressing Criteria B and C, conjecture remains surrounding appropriate cutoffs. Based on a recent study which had developed 95% confidence intervals for total (CI_95%_: 21.26_Mean_ ± 3.27) and child (CI_95%_: 4.26 _Mean_ ± 0.86) ADC scores using 47 healthy young Australian adults (93), participants who had met Criterion A whom also scored above the 95% confidence interval cutoff for the total (i.e., 25 or above) and child scores (i.e., 6 and above) were deemed to have met criteria B and C respectively. Twelve of the 13 participants who met Criterion A also met Criteria B and C. None of these participants reported a previous diagnosis of any neurological or medical condition affecting movement (e.g., cerebral palsy) and were deemed to have had intelligence at least in the normal range since they were recruited through the University setting and/or had completed an undergraduate degree (Criterion D). Finally, while 12 participants met the above criteria for DCD, only 8 met the required medical criteria to undergo TMS. Accordingly, our final sample of participants with DCD consisted of (*n*) 8. While no participant had a formal diagnosis of DCD, we can be confident that participants in our DCD group met criteria as outlined earlier. Using the same screening procedure, all controls were confirmed to have age-appropriate motor abilities, as indicated by a percentile ranking above the 20th percentile for “Total Motor Composite” scores of the BOT-2 [see also Ref. (83, 93, 100)] and were free of any self-reported medical or neurological impairments.

### Transcranial Magnetic Stimulation

Single-pulse TMS (Magstim-200 stimulator, Magstim Company Ltd., UK) was administered to M1 using a hand-held, 70 mm figure-of-eight coil that was positioned against the scalp using the orthodox method (handle pointing backwards and angled 45 ° away from midline). ppTMS was administered using the same coil and two-Magstim-200^2^ stimulators that were combined *via* a BiStim^2^ module. MEPs were recorded from the right-hand first dorsal interosseous (FDI) muscle using three EMG self-adhesive electrodes: an active electrode placed over the muscle belly of the FDI, a reference electrode over the interphalangeal joint of the right index finger and a ground electrode on the ulnar styloid process. For measurements of ISP, EMG was recorded from the FDI of the left hand, ipsilateral to the coil using LabChart v7 (66). EMG was acquired *via* a PowerLab/4SP system (AD Instruments, Colorado Springs, CO, USA). Grip force was measured *via* an AD instruments MLT004/ST grip force transducer.

Single-pulse TMS was used to locate the site of the M1 on the left hemisphere that would produce a maximal response in the right FDI. This “hotspot” location was marked and used as the site for TMS delivery for the rest of the session. Resting-motor threshold (RMT) was defined as the minimum intensity that produced a peak-to-peak MEP of >50 µV in at least 5 out of 10 consecutive trials (103). Active-motor threshold (AMT) was defined as the lowest stimulation intensity that, during tonic muscle contraction (~10% of maximal contraction as assessed *via* a grip force transducer), produced a peak-to-peak MEP of >200 µV in at least 5 out of 10 consecutive trials (104).

### Intrahemispheric Cortical Inhibition

Twenty baseline MEPs were then recorded following single-pulse TMS delivered at 4-s intervals, at 120% RMT. Cortical inhibition was then assessed *via* a number of ppTMS paradigms. Short-interval cortical inhibition (SICI) was recorded following the delivery of a subthreshold conditioning pulse (90% AMT), followed 2 ms later by a suprathreshold test pulse [120% RMT (105, 106)]. LICI was then recorded following the delivery of two suprathreshold pulses (120% RMT), separated by 100 ms (107–109). Both SICI and LICI were delivered at 4-s intervals. Ten recordings were taken for SICI and LICI, respectively. CSP was then recorded following single-pulse TMS (130% AMT) at 4-s inter-trial intervals (ITI) while participants maintained voluntary muscle contraction at ~20% of maximal voluntary contraction (MVC), as measured by the grip force transducer (61, 110). Fifteen recordings were taken for CSP.

### Interhemispheric Cortical Inhibition

Ipsilateral silent period and related EMG activity were recorded from the FDI of the left hand, following the delivery of 15 TMS pulses (150% RMT) to the ipsilateral hemisphere (111). Approximately 3 s before each pulse, participants were instructed to perform 100% of MVC of their left hand, by applying force with their index finger to the force transducer. The pulses were delivered with an ITI of 10 s, during which participants were instructed to relax their hand to prevent fatigue (112).

### Data Preparation

Motor-evoked potentials were analyzed by determining their peak-to-peak millivolt amplitudes. For SICI, each participant’s median conditioned peak-to-peak MEP was divided by their respective median unconditioned peak-to-peak MEP measured at baseline. The resulting value was then multiplied by 100 and subtracted from 100 to represent the percentage of inhibition of the test pulse [SICI (%)]

(1) $$\text{SICI }(\%)=100-\left[\left(\frac{C}{\text{NC}}\right)\times 100\right].$$

For LICI, each participant’s median peak-to-peak MEP in response to the second TMS pulse (the conditioned pulse) was divided by their median peak-to-peak MEP in response to the first TMS pulse (the non-conditioned pulse). The resulting value was then multiplied by 100 and subtracted from 100 to represent the percentage of inhibition of the second suprathreshold test pulse [LICI (%)]

(2) $$\text{LICI }(\%)=100-\left[\left(\frac{C}{\text{NC}}\right)\times 100\right].$$

For CSP, the onset and offset of the silent period was determined using the objective graphical method described by Garvey et al. (113). The CSP duration (CSP_duration_) was defined as the mean of the temporal difference (in ms) between CSP_onset_ and CSP_offset_ (CSP_duration_ = CSP_offset_ − CSP_onset_). Furthermore, since the duration of silent periods depend on stimulus intensities (61), the CSP_duration_ of each trial was divided by the peak-to-peak MEP amplitude of the same trial (CSP_ratio_) to reduce intersubject variability. The mean CSP_ratio_ was then calculated and used for subsequent analyses. Although not presented here, we note that the reported profile of between group comparisons for CSP did not differ appreciably without this normalization

(3) $${\text{CSP}}_{\text{ratio}}=\frac{{\text{CSP}}_{\text{duration in ms}}}{\text{peak}-\text{to}-\text{peak MEP amplitude}}.$$

The graphical method described earlier was also used to determine onset and offset for ISPs (113). The duration of ISP (ISP_duration_) was defined as the mean temporal difference between ISP_onset_ and ISP_offset_ (ISP_duration_ = ISP_offset_ − ISP_onset_). Given that the duration of ISPs also seems to be affected by stimulus intensities (61, 114), the ISP_duration_ of each trial was also divided by the peak-to-peak MEP amplitude evoked in the contralateral FDI of the same trial (ISP_ratio_). The mean ISP_ratio_ was then calculated and used for subsequent analyses. Although not presented here, we note that the reported profile of between group comparisons for ISP did not differ appreciably without this normalization

(4) $${\text{ISP}}_{\text{ratio}}=\frac{{\text{ISP}}_{\text{duration in ms}}}{\text{peak}-\text{to}-\text{peak MEP amplitude of contralateral FDI}}.$$

### Data Analysis

Group comparisons of intra- and interhemispheric cortical inhibition were conducted using Independent Samples *t*-tests for outcome measures where assumptions of normality were met (i.e., CSP_ratio_ and ISP_ratio_). Where assumptions were violated, Mann–Whitney *U*-tests were adopted [i.e., SICI (%) and LICI (%)]. Where significant group differences were observed, Pearson’s correlation analyses were conducted between those cortical inhibition measures and scale scores (M = 15, SD = 5) of relevant subtests from the BOT-2 requiring the use of the FDI. Since group differences were only observed on measures of interhemispheric cortical inhibition (see below), the purpose of the subsequent analysis was to determine whether this effect in DCD was associated with motor performance deficits. Only those subtests (i.e., “Manual Dexterity”) that placed greater demands on interhemispheric cortical inhibition (i.e., bimanual coordination of left and right hands) were subjected to correlations with our TMS measure of interhemispheric cortical inhibition.

## Results

All participants completed all measures. No group differences were observed for RMT or AMT. Baseline MEPs were also comparable between groups. As RMTs, AMTs and baseline MEPs were comparable between groups, and stimulator output percentages for SICI, LICI, CSP, and ISP are all based on RMT and AMT percentages, comparisons on stimulus intensities for SICI, LICI, CSP, and ISP were deemed redundant and were not conducted. See Table 1 below.

**Table 1:** Mean and SDs (in parentheses) of RMT and AMT and median baseline MEPs for DCD and TD groups.

	DCD	TD	*t*	df	*p*	Effect size (η^2^)
RMT (%)^a^	46.50 (9.17)	48.13 (6.85)	0.42	16	0.683	0.011
AMT (%)^a^	38.10 (8.39)	39.38 (6.37)	0.68	16	0.727	0.008
Baseline MEPs (mV)	1.10 (0.54)	1.27 (0.74)	0.18	16	0.858	0.002

*^a^Percentage of maximum stimulator output*.

*RMT, resting-motor threshold; AMT, active-motor threshold; DCD, developmental coordination disorder; TD, typically developing; MEPs, motor-evoked potentials*.

### Intrahemispheric Cortical Inhibition

**Figure 1:**
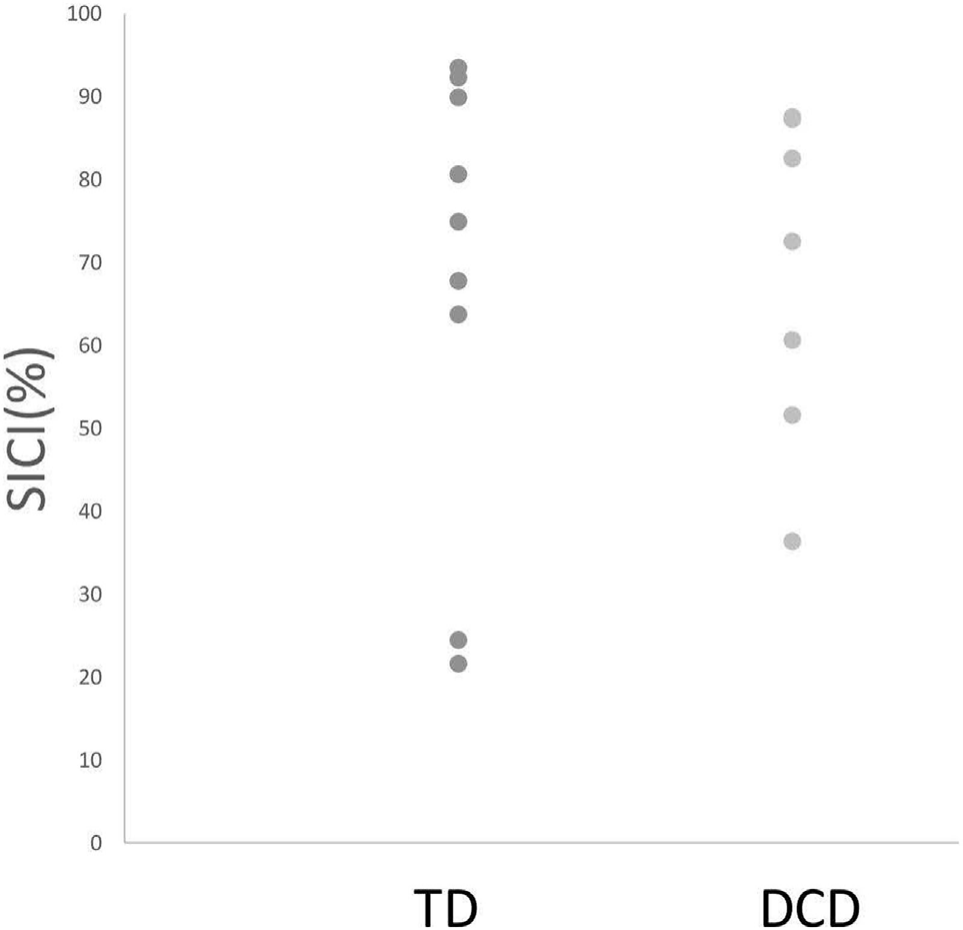
Median SICI (%) for both TD and DCD groups. Note: Due to the scale, one TD and one DCD participants are not shown in this figure because they demonstrated facilitation (and hence SICI % fell below 0). Abbreviations: SICI, short-interval cortical inhibition; TD, typically developing; DCD, developmental coordination disorder.

**Figure 2:**
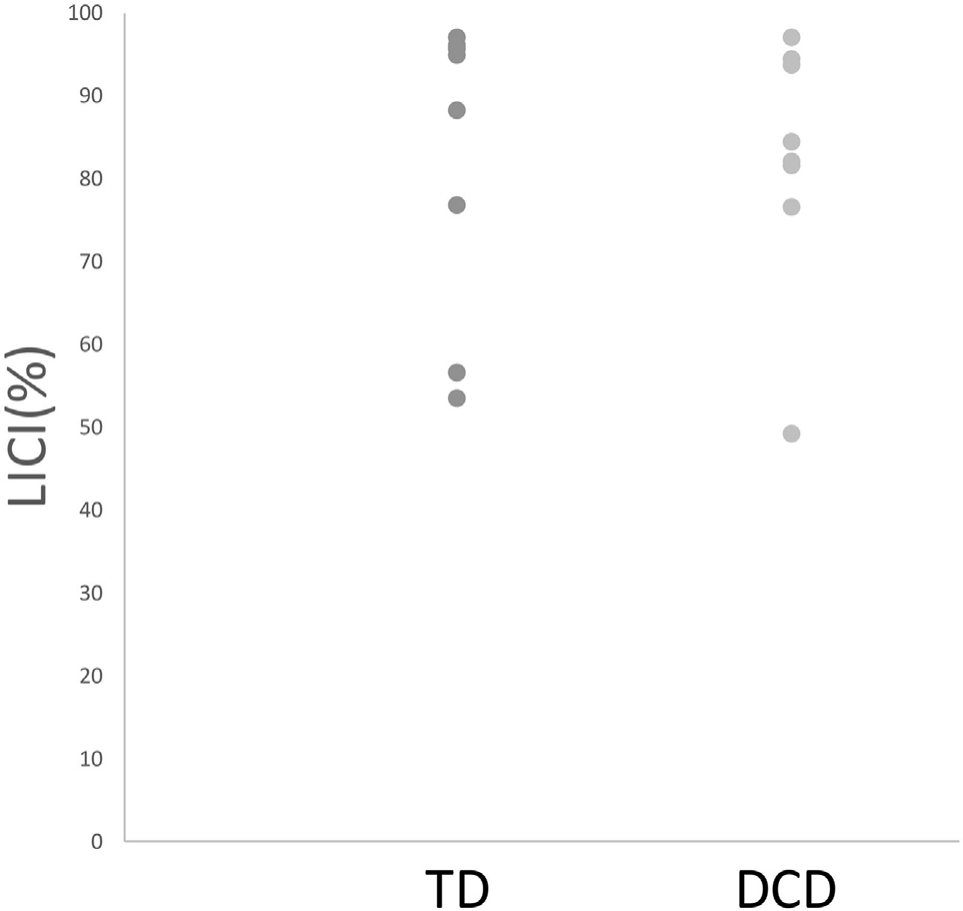
Median LICI (%) for both TD and DCD groups. Abbreviations: LICI, long-interval cortical inhibition; TD, typically developing; DCD, developmental coordination disorder.

**Figure 3:**
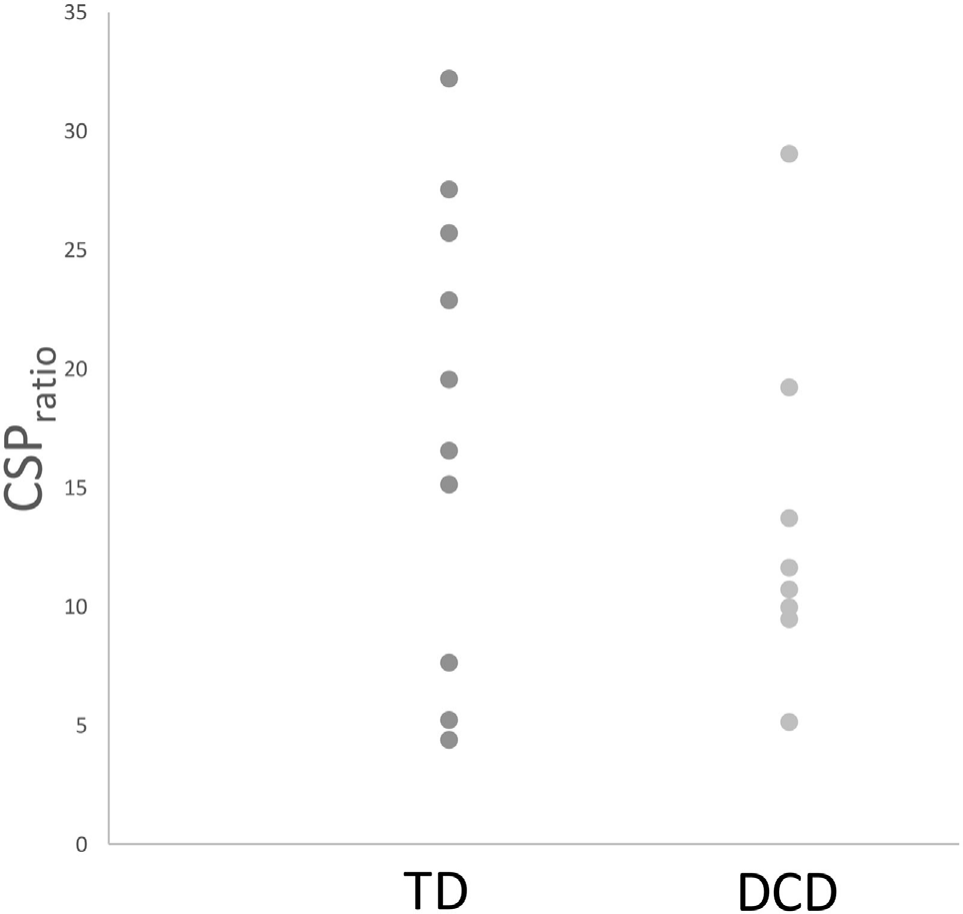
Mean CSP_ratio_ for both TD and DCD groups. Abbreviations: CSP, cortical silent period; TD, typically developing; DCD, developmental coordination disorder.

Comparisons of mean intrahemispheric cortical inhibition [SICI (%), LICI (%), and CSP_ratio_] found no significant differences between groups (see Table 2). Scatterplots for the intrahemispheric cortical inhibition measures are presented for DCD and TD groups in Figures 1–3 for SICI (%), LICI (%), and CSP_ratio_ respectively.

**Table 2:** Outcome measures for intrahemispheric cortical inhibition.

	DCD	TD	Statistic	df	*p*	Effect size
SICI (%)	54.80^Median^	73.51^Median^	30.00^U^	–	0.329	0.949^z^
LICI (%)	79.98^Median^	88.57^Median^	36.00^U^	–	0.648	0.738^z^
CSP_ratio_	13.61^Mean^ (7.41)	17.67^Mean^ (9.68)	0.978^t^	16	0.343	0.056^η2^

*^Mean^Group mean and SD in parentheses*.

*^Median^Group median*.

*^U^Compared using a Mann–Whitney U-test*.

*^t^Compared using an Independent Samples t-test*.

*^z^Kolmogorov–Smirnov Z*.

*^η2^Eta squared*.

*DCD, developmental coordination disorder; TD, typically developing; SICI, short-interval cortical inhibition; LICI, long-interval cortical inhibition; CSP, cortical silent period*.

### Interhemispheric Cortical Inhibition

**Figure 4:**
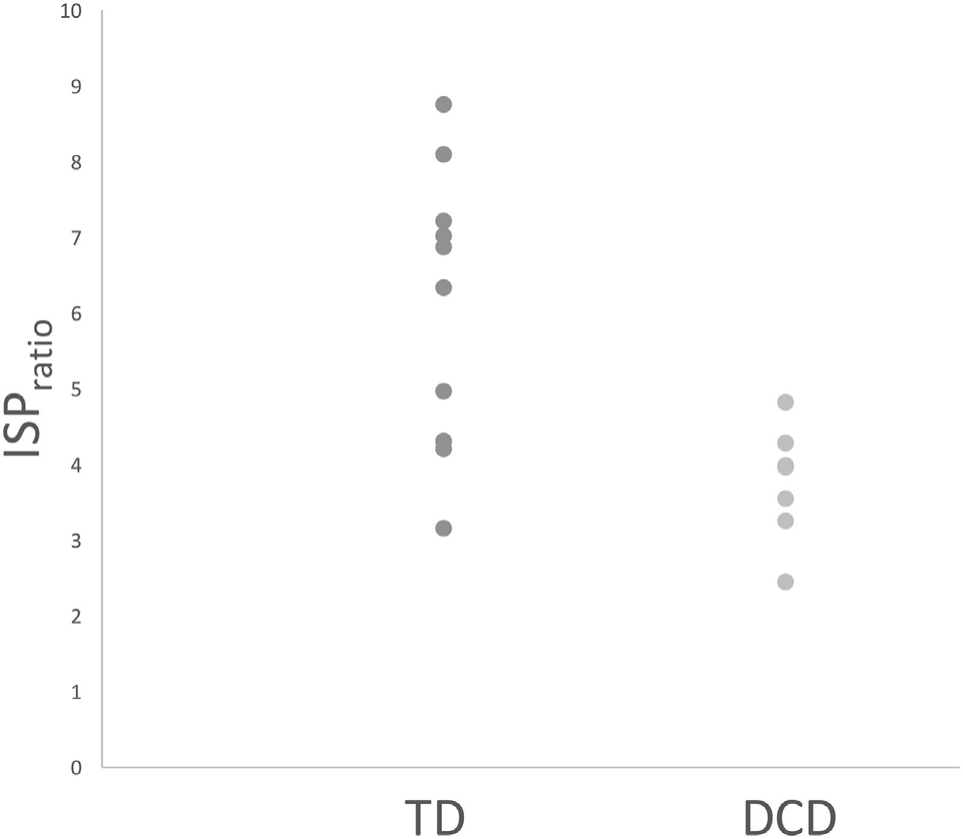
Mean ISP_ratios_ for TD and DCD groups. Abbreviations: ISP, ipsilateral silent period; TD, typically developing; DCD, developmental coordination disorder.

In regards to interhemispheric cortical inhibition, mean ISP_ratios_ were significantly smaller in the DCD group CI_95%_: 3.81_Mean_ ± 0.61 relative to TD controls CI_95%_: 6.08_Mean_ ± 1.32 (see Table 3). Individual mean ISP_ratios_ for participants in each group are shown in Figure 4. As can be observed, seven of eight DCD participants (or 88%) fell below the lower-bound CI_95%_ threshold of the TD group (i.e., 4.76).

**Table 3:** Mean and SD (in parentheses) of outcome measure for interhemispheric cortical inhibition.

	DCD	TD	*t*	df	*p*	Effect size (η^2^)
ISP_ratio_	3.81 (0.73)	6.08 (1.84)	3.56^a^	12.29	0.004	0.401

*^a^Violation of assumption of homogeneous variance was present, equal variances not assumed*.

*DCD, developmental coordination disorder; TD, typically developing; ISP, ipsilateral silent period*.

### Correlation Between ISP_ratios_ and BOT-2 Subtests

**Figure 5:**
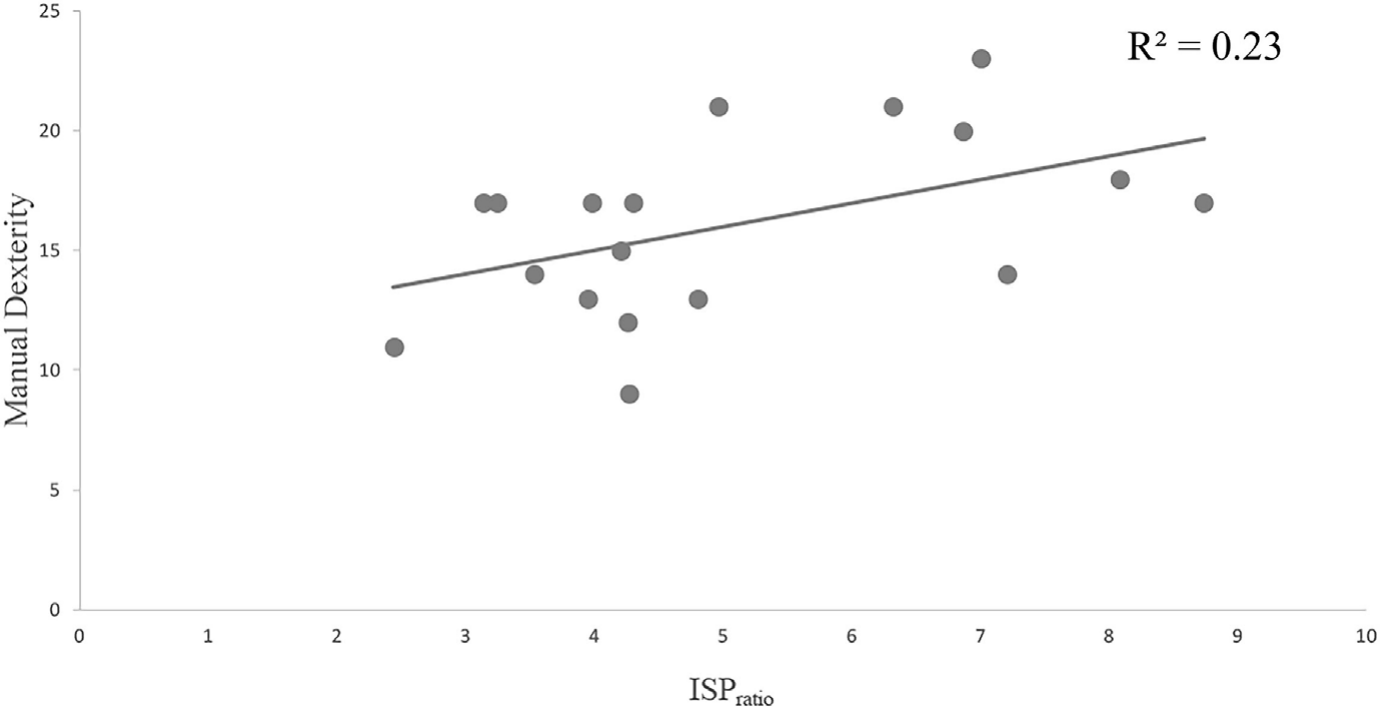
Pearson’s correlation between performance on the manual dexterity subtest from the BOT-2 and mean ISP_ratios_. Abbreviations: BOT-2, Bruininks–Oseretsky Test of Motor Proficiency, Second Edition; ISP, ipsilateral silent period.

Pearson’s correlation revealed a significant, medium to strong, positive correlation between mean ISP_ratios_ and “Manual Dexterity” scale scores of the BOT-2, *r* = 0.48, *n* = 18, and *p* = 0.044. A scatterplot illustrating this correlation is presented below in Figure 5.

## Discussion

The aim of this study was to investigate the integrity of intra- and interhemispheric cortical inhibition of the M1 in adults with DCD using a battery of single-pulse TMS and ppTMS protocols that are commonly implemented to measure these respective facets of cortical inhibition. Contrary to expectations, our results suggested that intrahemispheric cortical inhibition at rest is preserved in DCD, with no group differences observed on mean SICI, LICI, or CSP_ratios_. As predicted, however, we observed significant differences in mean ISP_ratios_ between groups, a commonly adopted measure of interhemispheric cortical inhibition. Indeed, adults with DCD showed significantly smaller mean ISP_ratios_ in comparison to healthy controls. Interestingly, the reduced interhemispheric cortical inhibition in the DCD group was also associated with poorer performance on subtests of the BOT-2 requiring bimanual coordination, shown by a significant correlation between mean ISP_ratios_ and performance on the BOT-2 manual dexterity subtest across groups.

### Intrahemispheric Cortical Inhibition Is Preserved in DCD

Based on behavioral findings indicating that individuals with DCD often display phenotypes consistent with reduced intrahemispheric cortical inhibition within the M1 (73–80, 84–87), we hypothesized that intrahemispheric cortical inhibition may be reduced in DCD. Contrary to expectations, comparisons between DCD and TD groups on a broad range of gold-standard indices of intrahemispheric cortical inhibition [i.e., SICI (%), LICI (%), and CSP_ratio_] failed to reveal statistically meaningful differences. This was particularly surprising given the behavioral phenotypes described earlier had been previously reported to rely on intrahemispheric inhibitory mechanisms within the M1 (25, 33). While it is possible that our modest sample size may have contributed to this lack of effect, we argue that the very small effect size (see Table 2) and considerable overlap in within-group variability across groups with and without DCD (see Figures 1–3) indicate that low study power is unlikely to account for our null findings. Thus, our results indicate that intrahemispheric cortical inhibition may be preserved in DCD, and, hence, is unlikely to be associated with previous reports of a decreased capacity to engage motor inhibition in this group as previously argued.

It is important to note that two of our measures of intrahemispheric cortical inhibition [i.e., SICI (%) and LICI (%)] were taken with the participant at rest (i.e., not performing an action) while the other (i.e., CSP) was taken while participants performed a very simple motor task (i.e., abduction of the FDI). Differences in resting-state cortical inhibition such as those adopted here have previously been detected between healthy controls and individuals with neurodevelopmental disorders where motor function is often impaired, such as ADHD (45). Furthermore, resting-state measures of intrahemispheric cortical inhibition have shown to predict motor competence in healthy individuals (44). Thus, we can be confident that measures of within hemisphere M1 cortical inhibition taken when the motor system is largely inactive (or at least not conducting complex movement) clearly provide important insight into the GABAergic properties of the motor system (44, 45, 115). These, it would seem, are preserved in individuals with DCD. However, resting-state measures such as those adopted here may not necessarily provide insight into how cortical inhibition within the M1 is modulated during fluent purposive action. As noted previously, successful inhibition of a prepared or prepotent action is typically preceded by an increase in cortical inhibition [as measured using SICI (25, 33)], supporting the view that modulation of cortical inhibition *on the fly* is central to the cancelation of movement [i.e., motor inhibition (19, 116)]. Thus, while the data from this study do suggest that the GABAergic processes within the inactive contralateral M1 may be preserved in DCD, it remains to be seen whether this group is able to modulate these processes flexibly during movement to support the suppression of unwanted movement, as is observed in healthy adults. Behaviorally, recent work has shown that children with DCD are less able to integrate inhibitory control with movements “online” to support corrections to movement mid-flight following unexpected perturbations to the reach target. Indeed, during double-step reaching tasks when the target of movements moves laterally following movement onset, children with DCD are more likely to complete movements toward a prepotent yet incorrect target before re-directing their action toward the new target [e.g., Ref. (78)], although reports of the effect are not constant [e.g., Ref. (84)]. Since inhibition of such movements requires active modulation of intracortical M1 inhibition (25), it is possible that this behavioral profile may be subserved by a decreased ability to modulate GABAergic systems flexibly during active movement. This remains speculative, however, and should be the focus of future work. Still, taken together, our data suggest that intrahemispheric cortical inhibition is preserved in DCD, at least when the motor system is not engaged in complex movement.

### Interhemispheric Cortical Inhibition Is Reduced in DCD

Comparison of ISP_ratios_ between DCD and TD groups found that the DCD group had significantly smaller ISP_ratios_ when compared with their TD counterparts. As discussed, ISPs are commonly used as a metric for indexing cortical inhibitory mechanisms involved in the suppression of unwanted transcallosal communication of motor commands across M1 hemispheres during lateralized movement (64, 66, 68, 117). It is a process thought to be particularly important for the accurate and efficient production of strict unimanual movements, and bimanual coordination where inhibition of homologous ipsilateral muscles is critical (35, 66). Thus, our finding that individuals with DCD had reduced ISP_ratios_ relative to controls supports the hypothesis that interhemispheric cortical inhibition of M1 activity may be atypical in individuals with DCD. Importantly, assessment of within-group differences showed that all but one participant in the DCD group (88%) fell below the 95% CI of the control group on mean ISP_ratios_. Thus, not only do those with DCD demonstrate decreased interhemispheric inhibition of M1 activity at a group level, but the effect appears to hold for a substantial proportion of individuals in this group. Furthermore, we observed that smaller ISP_ratios_ were associated with decreased performance on a subtest of the BOT-2 requiring bimanual coordination, and where the FDI was also used as the main effector (i.e., “Manual Dexterity”), with a compelling 30% of the variability in performance being explained by mean ISP_ratios_ (see Figure 5). These data are not only consistent with earlier accounts that interhemispheric inhibition of M1 activity is critical for accurate and efficient bimanual movements (118) but demonstrate that reduced interhemispheric M1 inhibition in DCD may be associated with their poor motor function (at least with respect to hand movement).

Furthermore, the reduced interhemispheric cortical inhibition identified in our DCD group is consistent with the growing number of behavioral studies which have consistently found more unwanted activity of ipsilateral homologous muscles (i.e., mirror movements) during lateralized unimanual motor tasks (76, 77) and bimanual coordination tasks (73, 74, 119) in DCD than TD controls. For example, during a recent finger tapping task which requires children to switch from bimanual to unimanual finger tapping, children with DCD made more additional taps of a non-cued finger when required to switch from tapping with both fingers to tapping with just one (119). Similarly, in a recent fMRI study, children with DCD displayed more unwanted activation of a non-cued hand during finger sequencing or fist clenching of the contralateral hand than their TD peers (77). However, despite the presence of such mirror movements, the authors of this particular fMRI study were unable to identify any activation deficits that could explain the presence of these unwanted actions. Interestingly a recent EEG study found that the increased incidence of mirror movements in DCD may be associated with reduced interhemispheric communication of inhibitory information (73). Specifically, children with DCD made more mirror movements during performance of a novel bimanual coordination task than their TD peers, with increased mirror movements correlating with lower cortico-cortical coherence between frontocentral regions of both hemispheres. Our neurophysiological findings are also in line with recent structural MRI work showing functional anisotropy reductions in calossal regions in children with DCD, the major communication pathway between hemispheres (120). While speculative, the decreased interhemispheric cortical inhibition of M1 activity observed here in DCD could certainly be a plausible mechanism with which to explain the reduced interhemispheric communication in individuals with DCD (73) and the greater mirror movements observed.

While the results of the present are promising, we acknowledge that we can only generalize these findings to the dominant M1 (left in this case), since cortical inhibition of the non-dominant hemisphere was not evaluated. That is, while our study shows that interhemispheric inhibition of right-hemispheric activity from the dominant left hemisphere is reduced in DCD, it cannot be assumed that this is also the case for the opposite hemisphere. Indeed, asymmetry in interhemispheric cortical inhibition has been noted in both patient groups with reduced ISPs (41) and controls (121). This issue remains an important avenue for future work, particularly given that excitability and inhibition within the M1 has previously been shown to be amenable to therapy (122, 123). Furthermore, as noted, by definition adults with DCD represent as a group of individuals for whom childhood motor difficulties persisted into adulthood. Thus, we must be mindful of generalizing the findings of our study to children with DCD not just because of the more obvious developmental factors, but also since any sample of children with DCD is likely to contain a subgroup of individuals whose motor difficulties dissipate with age and who thus represent a different subpopulation of individuals with motor difficulties to the one of interest here.

## Conclusion

This study was the first of its kind to examine the integrity of intra- and interhemispheric cortical inhibition in DCD using single-pulse TMS and ppTMS paradigms. While intrahemispheric cortical inhibition was comparable between DCD and TD groups, interhemispheric cortical inhibition was reduced in DCD. An additional correlation analysis conducted between ISP_ratios_ and the “Manual Dexterity” subtest of the BOT-2 found that TMS evoked ISPs were significantly related to performance on subtests requiring fine bimanual coordination. Taken together, our findings suggest that not only do individuals with DCD demonstrate reduced interhemispheric cortical inhibition of M1 activity, but that this may predict reduced motor performance in this group. Directions for future work and clinical implications were discussed. Our data are partly consistent with the view that cortical inhibition of M1 activity may be compromised in individuals with DCD and are consistent with behavioral accounts of reduced motor inhibition in this group.

## Ethics Statement

This study was carried out in accordance with the recommendations of Deakin University Human Research Ethics Committee (DUHREC) with informed consent from all subjects. All subjects gave written informed consent in accordance with the Declaration of Helsinki. The protocol was approved by DUHREC.

## Author Contributions

JH, CH, and IF contributed to all stages of this study, including project conception and design, data collection, analysis, and final write-up of the manuscript. PE and W-pT contributed substantially to project conceptualization and manuscript preparation. PB contributed to data collection and manuscript preparation.

## Conflict of Interest Statement

The authors declare that the research was conducted in the absence of any commercial or financial relationships that could be construed as a potential conflict of interest.
